# Multi-parametric analysis of speech timing in inter-talker identical twin pairs and cross-pair comparisons: Some forensic implications

**DOI:** 10.1371/journal.pone.0262800

**Published:** 2022-01-21

**Authors:** Julio Cesar Cavalcanti, Anders Eriksson, Plinio A. Barbosa

**Affiliations:** 1 Department of linguistics, Stockholm University, Stockholm, Sweden; 2 Institute of Language Studies, Campinas State University, Campinas, Brazil; Universite Sorbonne Nouvelle Paris 3, FRANCE

## Abstract

The purpose of this study was to assess the speaker-discriminatory potential of a set of speech timing parameters while probing their suitability for forensic speaker comparison applications. The recordings comprised of spontaneous dialogues between twin pairs through mobile phones while being directly recorded with professional headset microphones. Speaker comparisons were performed with twins speakers engaged in a dialogue (i.e., intra-twin pairs) and among all subjects (i.e., cross-twin pairs). The participants were 20 Brazilian Portuguese speakers, ten male identical twin pairs from the same dialectal area. A set of 11 speech timing parameters was extracted and analyzed, including speech rate, articulation rate, syllable duration (V-V unit), vowel duration, and pause duration. Three system performance estimates were considered for assessing the suitability of the parameters for speaker comparison purposes, namely global Cllr, EER, and AUC values. These were interpreted while also taking into consideration the analysis of effect sizes. Overall, speech rate and articulation rate were found the most reliable parameters, displaying the largest effect sizes for the factor “speaker” and the best system performance outcomes, namely lowest Cllr, EER, and highest AUC values. Conversely, smaller effect sizes were found for the other parameters, which is compatible with a lower explanatory potential of the speaker identity on the duration of such units and a possibly higher linguistic control regarding their temporal variation. In addition, there was a tendency for speech timing estimates based on larger temporal intervals to present larger effect sizes and better speaker-discriminatory performance. Finally, identical twin pairs were found remarkably similar in their speech temporal patterns at the macro and micro levels while engaging in a dialogue, resulting in poor system discriminatory performance. Possible underlying factors for such a striking convergence in identical twins’ speech timing patterns are presented and discussed.

## Introduction

The present study set out to assess the speaker-discriminatory potential of a set of speech timing parameters in comparisons performed between identical twin pairs (while engaging in dialogue) and in cross-pair comparisons. A multi-parametric analysis was conducted, comprised of the assessment of macro, micro, and pause-related speech timing estimates, including those commonly assessed within the forensic speaker comparison domain (e.g., speech rate and articulation rate).

The primary motivation for including the comparison of identical twins in the present study lies in the fact that such individuals represent an extrapolation of the highest possible similarity between subjects, both from a physical and sociolinguistic point of view, allowing the assessment and understanding of inter-subject variation levels.

In terms of structural or anatomical characteristics, identical twins are assumed to have very similar vocal tracts in size and shape, as studies with genetically identical speakers support the genetic makeup of an individual as a major factor in determining his or her overall size, shape, rate of growth and maturation [1]. Furthermore, genetically identical twins have been suggested as almost entirely correlated in their gray matter distribution, including areas related to language cortices, as observed by [2] with brain imaging. Another important observation is that identical twins raised together were exposed to very similar stimuli during their language acquisition and development, which may unquestionably impact their linguistic patterns.

As remarked by [3], investigations on the speech patterns of twins from a forensic phonetic perspective allow researchers to understand the very limits of variation between speakers. According to the author, if differences in speech parameters can still be found when common sources of inter-speaker variation are substantially reduced, then the potential forensic application of such parameters increases.

It is worth noting that, as far as twin studies are concerned, very little research has been carried out on the speech timing domain and that small-sized experiments are persistent, cf. [4], contrasting from studies on other acoustic domains, as in the analysis of glottal source features, cf. [5, 6]. Such observations justifies the analyses conducted here.

## Background

The concept of time is inherent to the description of any dynamic system, which also extends to the realm of speech production and perception. Notably, duration patterns can be identified in many linguistic organization levels and are systematically exploited by languages when implementing contrast [7], from the segment, passing by the syllable up to higher linguistic domains. In that regard, “timing”, as described by the organization of duration throughout the utterances, can be assessed at different linguistic levels depending on the researcher’s interest.

Apart from widely acknowledged general linguistic temporal patterns, how do individuals vary in speech timing measures when speaking in the same language and dialect? Can such a variation, within limits imposed by the production system, be regarded as speaker-discriminatory? Moreover, what are the effects of reducing common sources of inter-speaker variation on speech temporal patterns? The present study represents an attempt at addressing such questions with special consideration to spontaneous speech materials. To this end, some methodological aspects are revisited and developed in the following.

### Methodological considerations

Measuring speech tempo from a signal-based approach requires some methodological criteria to be considered and some experimental decisions to be made. As pointed out by [8], the primary decision concerns the linguistic unit based on which the parameter will be estimated, namely *the unit of measurement*. This could be segments per second, syllables per second, words per second. According to the author, although there are arguments for all of these choices, the most commonly used unit is the syllable, especially in the domain of forensic speaker analysis. Secondly, it must be decided which linguistic dimension the estimations will be based on, either based on the “canonical” (abstract) or realized (concrete) units. It is worth mentioning that, depending on the dimension chosen, different estimations may be obtained; as syllable reductions are reasonably common in spoken corpora, an analysis on the basis of produced syllables, for instance, often tends to result in lower but articulatory more faithful speaking rate estimates.

A third methodological aspect mentioned in [8] regards the size and kind of speech unit used for the analysis. Concerning this aspect, estimations can be made over the entire duration of a recording, yielding a global measure expected to portray a speaker’s habitual temporal speech behavior, or over smaller portions throughout the recording, expected to capture local temporal variations that may be relevant for the analysis. Notably, the *size* and kind of *speech units* adopted also have practical consequences, as verified in the present study. Smaller units or units that are considerably more frequent tend to yield higher amounts of data, which, in practice, may enhance the statistical power of the analysis being performed, given that statistical models are unquestionably sensitive to the variable’s number of observations [9]. In that regard, an extraction based on syllables or vowel segments naturally tend to result in more data points than measures extracted from longer speech units, such as words or intonation phrases.

In the present study, a syllable-sized duration unit named V-V unit (i.e., vowel-to-vowel unit) was adopted, based on which speech rate, articulation rate, and syllable duration were assessed. The relevance of this unit in psychoacoustic terms is broadly discussed in [10], and its explanatory potential of the speech rhythm production explored in [11]. Such a phonetic unit comprises all the segments uttered between two consecutive vowel onsets, with the onset of the following vowel defining the beginning of a new V-V unit. It has been studied and employed among others by [11–14], with its application tracing back at least to [15, 16].

Finally, adding to the before-mentioned criteria, another crucial aspect regards the treatment given to pauses. Notably, the inclusion or exclusion of pauses (i.e., silent and filled pauses) in the speaking rate estimations may yield different outcomes. Electing one parameter over the other should be motivated by what is being analyzed and the research goal. Moreover, this fundamental difference contrasts two of the most commonly used speech tempo parameters, namely speech rate, when pauses are kept in the intervals, and articulation rate, when pause duration is not included when calculating the total sample duration [17, 18]. Regardless of whether silent pauses should be included or excluded in the analysis, their minimum length must be defined and controlled to prevent the inclusion or exclusion of silent intervals that are not related to pausing behavior (e.g., silent closure periods in the acoustic signal). As remarked by [17], based on the findings in the literature concerning automatic measurements, a threshold value of 100 ms appears adequate in order to prevent counting occlusion phases of plosives as silent pauses.

### Tempo in speech: Aspects of production

The observation that speakers vary regarding their speech tempo patterns is commonplace. In this respect, many factors, from different orders, are known to account for such variability, as in the case of linguistic and extra-linguistic factors. Some of these are speaking style [19, 20], dialect [20], phrase length [21], age [20–22], sex [20], the emotional state of speakers [23], neuromuscular and sociolinguistic factors [18, 24]. Other factors within the speech pathology domain have also been suggested, such as cognitive decline [25] and speech-language disorders (e.g., stuttering disorder) [26].

In the study conducted by [19], speaking style has been found to significantly affect the produced speech rate, articulation rate, frequency of pauses, pause ratio, mean pause duration, and the standard deviation of pause duration within speakers. Speech and articulation rates were lower in retelling (text recall) when compared to other speaking styles, such as reading and spontaneous conversation. Furthermore, pauses tended to be more frequent and also longer for the retelling condition. The highest speech and articulation rates and the shortest pauses were observed for the reading style.

In an experiment conducted by [20], the researchers also found significant differences when comparing articulation rate in spontaneous unconstrained talks and sentence reading. The results showed that speakers who had a faster speaking rate also had a faster reading rate. When the reading rate increased by one syllable per second, speaking rate increased by 0.69 syllables per second. According to the authors, the outcomes reveal a relationship between the articulation rate in speaking and in reading, which may suggest the existence of the same underlying motor control mechanism for speaker-specific rate.

Concerning the variables sex and dialect, [20] noted that males tended to speak significantly faster than females in an experimental study. However, this difference was only significant in spontaneous speech, not being verified for reading rate. Furthermore, although a speech tempo difference between males and females was present, it was smaller than observed in a cross-dialect comparison, namely American English spoken in Wisconsin and North Carolina. In general, Wisconsin speakers displayed a significantly faster speaking rate than North Carolina speakers. Even though the referred study has not been carried out within a forensic-phonetic frame, such a finding may indicate the relevance of considering the dialect impact when comparing individuals from different populations or subjects who have potentially migrated to different dialectal areas.

As previously mentioned, variation in speech timing characteristics may also be explained as a function of linguistic factors. From this perspective, considerable attention has been paid to phrase length effects on speech tempo characteristics.

In the study conducted by [21] with Dutch speakers (school teachers), articulation rate was analyzed in a spontaneous speech corpus employing a multilevel/mixed-effects modeling including explanatory factors such as the speaker’s sex, age, country of origin, dialectal region, and phrase length. The outcomes of the referred study revealed that speech tempo appears to be partly determined by phrase length, which according to the researcher, is possibly due to a mechanism known as “anticipatory shortening”. This mechanism appears to account for why longer phrases, containing more syllables, tended to be spoken at a faster rate and shorter average syllable duration.

Notwithstanding, it is noteworthy that the existence of such a “anticipatory shortening” mechanism is disputed. Contrary to what was observed by [20, 21] found evidence for shorter phrases containing fewer syllables to be spoken faster in an experiment with American English speakers. According to [20], one possible explanation for such a cross-study divergence may relate to the fact that while participants in the Dutch corpus were school teachers (i.e., who possibly have, by practice, a better command of spoken language in terms of articulatory planning, verbal monitoring, and effective use of pauses), American English speakers varied regarding their professional and educational background. Consequently, American English speakers would also be expected to vary in their experience with spoken language usage.

Regarding the present study, it is worth mentioning that those variables acknowledged in the literature as bearing influence on speech timing measures, such as age, sex, speaking style, and dialect, may be regarded as relatively controlled, considering that only adult young male individuals from the same dialectal region were recruited. Moreover, all individuals were recorded in the same speaking style: a spontaneous telephone conversation. The possible effects of phrase length on the temporal measures are also minimized since representative data were used (i.e., spontaneous speech material), containing many possible realizations from each speaker, extracted from different dialogue parts in the recordings. Finally, the familiarity effect between speakers, a very often neglected factor, can also be regarded as controlled, given the fact that all recordings were performed while twin speakers interacted with each other.

### Forensic-phonetic studies on speech timing

Despite the relevance of temporal parameters for forensic speaker comparison purposes, for example due to their relative resistance concerning the limitations imposed by the telephone transmission system, cf. [27, 28], very few studies have been conducted with temporal measures from this perspective. Furthermore, very little is known about the effects of a shared linguistic environment and the similarity between individuals in establishing individual timing patterns. This research gap motivates and justifies the relevance of the present study. In order to provide an appropriate context, some of the relevant studies available on speech timing analysis within the forensic perspective are furthermore rehearsed.

A pioneering study was conducted by [17] within the forensic phonetic domain with five male and five female German speakers, aged between 20 and 26. The experiment set out to assess the speaker-specific potential of speech timing parameters under three different speaking styles and two forensic-related recording conditions. The referred styles were spontaneous speech, semi-spontaneous speech (i.e., a conversation recall), and reading. The recording conditions were “direct” recording and “telephone recording”. Overall, seven speech timing parameters were computed and analyzed, namely speech rate (i.e., syllable rate), articulation rate, amount of pause activity, pause-free intervals (i.e., the average time span between pauses), the average number of syllables produced between pauses, the ratio of silent and filled pauses, and the ratio of pauses with and without respiratory activity. While non-significant differences were observed between “spontaneous” and “semi-spontaneous” speech, differences between these two speaking styles and “reading” were most significant, except for articulation rate. In general, the read speech was characterized by higher speech rates, fewer and shorter pauses, fewer hesitations, a larger number of respiratory pauses, and longer inter-pausal intervals. Regarding the comparison between telephone transmitted speech and face-to-face speech, no significant differences were observed for most speaking tempo parameters, except for an increase in the proportion of filled pauses in the telephone transmitted speech. According to [17], the higher occurrence of filled pauses in the telephone condition may be explained as the speakers’ attempt to signal their intent to keep their turn to an interlocutor who cannot be addressed visually by gestures or facial expression. In addition, according to the same study, the findings suggest the measure of articulation rate as remarkably constant within speakers, and therefore, a promising speaker-specific parameter for forensic speaker comparison.

In the same perspective, a comprehensive study on articulation rate was carried out by [8] with a group of 100 German-speaking male subjects, ranging between 21 and 63 years of age (average of 39 years). Articulation rate was assessed in three different conditions: face-to-face spontaneous speech, spontaneous speech over the telephone, and reading. The speech context in the first two conditions was a descriptive task (i.e., the description of a set of pictures to a conversation partner). The analysis of articulation rate was carried out globally and locally based on phonetic syllables. The speech unit chosen for the extraction of the measures was “memory stretch”, characterized by portions of fluent speech containing a number of syllables that can easily be retained in short-term memory. Global articulation rate was computed as a function of mean articulation rate across stretches, including standard deviation estimates. The threshold in a memory stretch ranged from 4 to 20 syllables, excluding filled pauses, silent pauses, and syllable lengthening. Regarding the outcomes, contrary to what was reported by [17], it was found that both direct and telephone-transmitted speech deviated significantly from reading, as expressed by an increase in the parameter for the reading style. Regarding the analysis of standard deviation values, direct speech and telephone-transmitted speech were again found distinct from reading, in which a lower standard deviation was observed for the latter. In addition, similarly to [17], no significant differences were observed between direct and telephone-transmitted spontaneous speech. Finally, according to [8], the observation that intra-speaker variability across reading and spontaneous speech was greater for articulation rate standard deviation than for its mean suggests the analysis of mean articulation rate as more viable from a forensic viewpoint. According to the author of [8], such knowledge could be applied to compensate for a speaking style mismatch and considered when guidelines for forensic speaker comparisons are proposed.

As for Brazilian Portuguese, a phonetic experiment was conducted by [13] on speech tempo parameters based on a realistic forensic data-set. The analyzed material comprised of spontaneous speech samples derived from intercepted telephone conversations and direct non-contemporaneous recordings of the same speakers. Seven speakers were analyzed, namely five males and two females, aged between 14 and 31 years (mean of 24 years) in the first speech sample and between 15 to 33 years (mean of 26 years) in the second condition. Global and local speech rate and articulation rate were assessed inter- and intra-subjects. Following a general expected trend, higher values were obtained for articulation rate in comparison to speech rate. Although non-statistically significant, higher variability was observed for speech rate in both global and local measurements. Regarding the measurement procedure (i.e., global and local measurements), significant differences were observed between global and local measures only for speech rate, whereas for articulation rate, both global and local measurement methods yielded similar outcomes. When assessing the variance of the parameters through F-tests, it was found that although local measurements of speech and articulation rates tended to display less variation, no statistical significance between the local and global measures was observed. Concerning the intra- and inter-speaker variability levels, an intra-class correlation coefficient analysis (ICC) suggested that only articulation rate (global and local measures) fulfilled the requirement of a higher inter- than intra-speaker variation.

Furthermore, the study conducted by [12] with 35 male Brazilian Portuguese speakers from seven different regions in Brazil aimed at assessing the speaker- and dialect-discriminatory power of eight acoustic parameters, including speech rate analysis, in different harmonic-to-noise ratios. The addition of different signal to noise (S/N) levels (0.01 and 0.02 dB) to the recordings intended to evaluate the parameters’ robustness regarding a prevalent type of audio degradation in forensic-related conditions. In the experiment, both spectral emphasis and the median speech fundamental frequency were affected by the Gaussian noise addition. The results also revealed a more abrupt change in the spectral emphasis than for the F0 median, reaching an increase of 55% (Gaussian 0.01) and 154% (Gaussian 0.02) in relation to the original recording. As for the F0 median, the greatest change was 3 Hz, which, despite being statistically significant, possibly does not interfere in the discrimination of a subject, as remarked by the researchers. Most importantly, the outcomes of the referred study suggested the analysis of speech rhythm-related parameters (e.g., speech rate and V-V unit duration) as the most consistent approach when dealing with audio samples containing noise distortions.

The findings deriving from [8, 12] have important implications for the forensic speaker comparison practice since they signal the relative resistance of speech tempo measures to variables commonly present in forensic casework, serving as an adequate alternative to situations where other parameters (e.g., vowel formant analysis in telephone recordings) are not reliable. Despite the relevance of assessing the temporal dimension of speech from a forensic phonetic perspective, few studies have addressed the speaker-discriminatory potential speech timing estimates. Such a research gap is especially true when considering spontaneous speech-oriented studies or experiments with very similar speakers, e.g., identical twin pairs. The present study represents an attempt at advancing such understanding.

## Research questions and hypothesis

The following research questions were addressed in the present study:

- Which set of speech timing parameters are considerably speaker-discriminatory and therefore suitable for the forensic speaker comparison application?
- Which speech temporal dimension(s), namely, macro, micro, and pause-related, can best explain individual-related patterns?
- Is it possible to differentiate identical twins through the assessment of their speech timing measures even when they are engaged in a conversation?

Although intra-twin comparisons are expected to reveal a great deal of similarities—perhaps potentialized by different levels of prosodic entrainment, some twin pairs may still be found phonetically different, suggesting the influence of “choice” regarding their speech timing patterns. The “choice” influence, as a potential factor accounting for intra-twin pair differences, has been suggested by a previous study conducted with the same group of speakers at the vowel formant frequency domain, cf. [29], as well as by other studies, cf. [3, 30–33].

## Materials and methods

The present study registered under the protocol 95127418.7.0000.8142 was evaluated and approved by the ethical committee at Campinas State University (UNICAMP). All participants voluntarily agreed to be part of the research verbally and by signing a participant consent form. All personal information regarding the participants is kept private.

### Participants

The participants are 20 subjects, ten male identical twin pairs, Brazilian Portuguese (BP) speakers from the same dialectal area. The participants’ age ranged between 19 and 35 years, with a mean of 26.4 years. All identical twin pairs were assigned a letter and a number, according to the following pattern: A1, A2, B1, B2, C1, C2, D1. The same speaker letters were used to indicate identical twin individuals.

The decision to adopt the term “identical twins” over “monozygotic twins” resides on a practical reason: the latter terminology implies assessing the twins’ genetic material. As no laboratory genetic assessment was carried out, the first term will be preferred. However, it is worth noting that the expression “identical” does not imply that speakers are identical to each other, based solely on the relatively high physical similarities displayed by the twin pairs.

Speakers were recruited through a recruitment method known as chain sampling or “snowball”, in which subjects are contacted among their acquaintances or by recommendation of other participants of the study. Each twin within the pair lived and resided in the same city/town. The pairs were recruited in five different cities in the state of Alagoas, which stands for the second smallest state in Brazil.

The inclusion criteria were: i. Identical twins; ii. male speakers; iii. same dialect; iv. aged between 18-45 years; v. with at least elementary school completed. The exclusion criteria were: i. Reported hearing loss or speech disorder, ii. identical twins raised apart; iii. identical twins that lived apart from each other for more than five years.

All twin pairs in the present study were raised together, studied together, were frequently in contact with each other, and displayed a high-affinity level. For that reason, the familiarity effect between twin pairs can be regarded as relatively controlled.

### Recordings

The recordings were carried out in silent rooms located in the cities where the twins resided. The speech material used in the present research consists of spontaneous telephone conversations between twins, with dialogue topics being decided by the pairs, aiming at elucidating more ecologically valid material. During the recording sessions, twin pairs were placed in different rooms, not directly seeing, hearing, or interacting with each other. The speakers were encouraged to start a conversation using a mobile phone while being simultaneously recorded through high-quality microphones. The audio signals were then processed and registered in two separate channels. Such a recording approach aimed at eliciting a telephone speaking style and represents an attempt to approximate the experimental conditions to more realistic forensic circumstances, as conducted in [6].

All recordings were carried out with a sample rate of 44.1 kHz and 16-bit amplitude resolution, using an external audio card (Focusrite Scarlett 2i2) and two headset condenser microphones (DPA 4066-B). The unedited recordings had an average duration of about 10 minutes. In all cases, the conversation topics were decided by the twins beforehand during the recording sessions.

### Data transcription and extraction

In total, a set of 11 temporal speech parameters were analyzed, including macro, micro, and pause-related temporal parameters, as described below. All parameters were extracted automatically using the Praat script *ProsodyDescriptorExtractor*, cf. [34].

Because it was logical from a practical viewpoint, the studied parameters were classified and grouped into three main categories: macro, micro, and pause-related temporal parameters. Such a classification followed a duration criterion, namely the duration of the phonetic syllable, i.e., the V-V unit. Such a division is expected to help reporting of the outcomes and the discussions.

The first category includes those parameters extracted from units with an average duration superior to that of the phonetic syllable, namely speech rate, articulation rate I, articulation rate II, and stress groups. The second category includes those parameters extracted from units with a mean duration equal to/below the syllable duration, which includes V-V units and vowel segments. Finally, all pause-related parameters were grouped in the same category, namely silent pauses, filled pauses, all pauses, and inter-pausal intervals.

**Macro speech timing parameters**:

- Speech rate (SRATE): defined as the number of V-V units in each speech chunk divided by its total duration (V-V units/seconds), including silent and filled pauses.
- Articulation rate I (ARTRATE I): defined as the number of V-V units in each speech chunk divided by its total duration (V-V units/seconds), excluding only silent pauses.
- Articulation rate II (ARTRATE II): defined as the number of V-V units contained in each speech chunk divided by its total duration (V-V units/seconds), excluding silent pauses and lengthened vowels.
- Stress group duration (SGDUR): defined as the interval corresponding to two consecutive salient V-V units (in seconds), i.e., those units for which a duration increase has been automatically detected. Each stress group ends with a salient V-V unit.

**Micro speech timing parameters**:

- V-V units duration (VVDUR I): syllable-sized units defined as all the segments uttered between two consecutive vowel onsets (in milliseconds). Both salient and non-salient V-V units are included in this parameter.
- V-V units duration (VVDUR II): the aforementioned phonetic unit corresponding solely to non-salient V-V units (in milliseconds), represented by those units for which a duration increase has not been automatically detected.
- Vowel duration (VOWEL DUR): defined as the duration of produced oral monophthongs (in millisecond).

**Pause-related parameters**:

- Silent pauses duration (SILPAUSES): silent pauses (in milliseconds) equal or superior to 100 ms, a threshold commonly applied in automatic measurements, as to prevent occlusion phases of plosives from being counted, cf. [17].
- Filled pauses (FILPAUSES): defined as vowel prolongations equal or superior to 100 ms, perceived as hesitations/filled pauses (in milliseconds).
- All pauses (ALLPAUSES): combination of silent and filled pauses (in milliseconds), i.e., lengthened vowels before silent pauses or in hesitations.
- Inter-silent pauses intervals (IPI): defined as the interval comprising the speech production between two consecutive silent pauses (in seconds).

**Fig 1:**
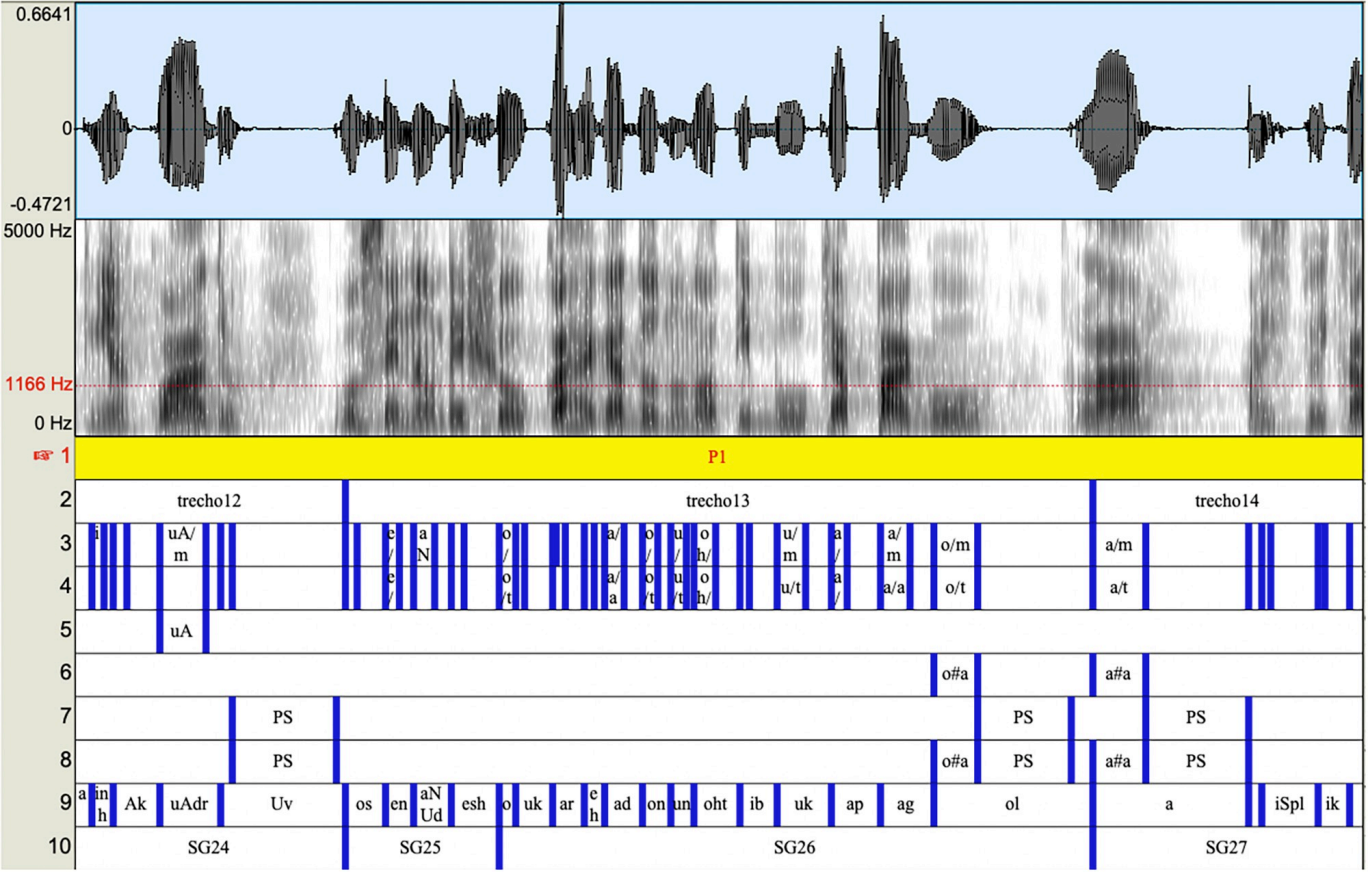
Data segmentation and annotation.

The data segmentation and transcription were performed manually in the Praat software [35]. All data were transcribed and reviewed by the first author, who is a phonetician and a certified speech-language pathologist, as well as a native speaker of the dialect studied. Fig 1 visualizes how speech units were grouped into 11 different layers in the Praat textgrid, as follows:

1. **Dialogue part**: different portions/parts of the dialogues throughout the recordings, e.g., beginning, middle, and final parts;
2. **Speech chunks**: speech intervals, in most cases corresponding to inter-pause intervals (i.e., stretches of speech between long silent or filled pauses);
3. **All vocalic segments**: all vocalic segments produced within speech chunks, including monophthongs, diphthongs, and nasalized vowels;
4. **Oral monophthongs**: oral monophthongs only;
5. **Oral diphthongs**: oral diphthongs only;
6. **Filled pauses**: vowel prolongations with a minimum duration threshold of 100 ms;
7. **Silent pauses**: silent pauses with a minimum duration threshold of 100 ms;
8. **All pauses**: combination of silent and filled pauses;
9. **Vowel-to-vowel units**: syllable-sized duration units defined as all the segments uttered between two consecutive vowel onsets;
10. **Smoothed z-scores peak values**: smoothed z-scores peak values at the end of the stress group (generated semi-automatically, cf. [34]);
11. **Stress groups**: intervals corresponding to two consecutive salient V-V units (generated semi-automatically, cf. [34]).

The durations of the selected speech chunks (see *layer 2* of the Praat textgrid in Fig 1) were around 3 s as an attempt to match “speech turn time” variation among speakers. There was an already expected tendency for some subjects to hold their turn for a longer time than others. Furthermore, inter-pause intervals were tracked throughout the transcriptions and used in most cases as a more objective criterion for segmenting chunks (i.e., intervals between longer pauses, never containing less than three V-V units). As for longer intervals, without perceived silent or filled pauses, these were preserved in their total duration, or in some cases, divided into smaller parts while maintaining the structure of intonational phrases. Moreover, considerable agreement regarding chunk boundaries and stress group boundaries was observed.

By the end of the transcription process, individual speech chunks were precisely, on average, 3.12 s long. Note that this value corresponds to the right limit of 95% confidence intervals for stress group duration in Brazilian Portuguese, cf. [36]. The average V-V unit count in each speech chunk was 9.9 units, with a minimum of 3 and a maximum of 32 units, with 14 units being the most recurrent number. The minimum of 3 V-V units criterion aimed to prevent the selection of speech chunks under the effects of phrase-final lengthening. The 3 V-V units threshold was set to avoid the inclusion of very short speech chunks that could increase the effect of phrase-final lengthening on the calculated parameters.

Vowel segments were segmented and transcribed manually following auditory and acoustic criteria, namely the careful listening to the speech stretches and the observation of the energy appearance/disappearance in the broad-band spectrogram. After the segmentation of all vowels in *layer 3* of the Praat textgrid, oral monophthongs were segmented in a separate textgrid layer (see *layer 4* in Fig 1), from which nasalized vowels, diphthongs, and triphthongs were disregarded. Oral monophthongs were then manually classified as stressed or unstressed for the purpose of the analyses performed in a previous study, cf. [29]. The analysis of diphthongs in *layer 5* remains a topic for future investigation.

The main acoustic criterion for segmenting silent and filled pauses (*layers 6* and *7*, respectively) was their relative duration. Only silent pauses and vowel lengthening with a minimum duration of 100 ms were included for the comparisons. Breathing sounds, as in the case of inhalation noises, were included in the non-speech part. The referred threshold was established based on the observation that most pauses produced by the speakers exceeded this limit, and also in light of previous studies [17]. As for filled pauses (vowel lengthening), for the sake of parametrization, the same threshold was applied. As such, perceived silent pauses and lengthened vowels below this limit were excluded from the tested data-set. Frequently, there was some uncertainty whether a vowel lengthening corresponded to a filled pause or a prosodic emphasis. Such occurrences were not included in the present data set. Finally, both silent and filled pauses were combined in *layer 8*, as to allow the computation of articulation rate II.

Following the segmentation of all vowels in *layer 3* in Fig 1, V-V units were manually transcribed (*layer 9*). Such a segmentation is what the script *ProsodyDescriptorExtractor*, cf. [34], requires to generate the smoothed z-scores peak values and stress groups (*layers 10* and *11*, respectively).

### Statistical analysis

All statistical analyses were carried out in the R software. As most of the data in the present study do not meet the normal distribution requirement, as verified through the Shapiro-Wilk normality test (*p* < 0.05), the statistical testing was performed by means of non-parametric methods. The Kruskal-Wallis rank-sum test was applied to verify possible differences in each tested parameter, followed by the post hoc analysis with the Dunn’s Multiple Comparison Test (two-tailed). The Bonferroni correction was automatically performed to adjust the alpha threshold due to multiple comparisons, based on the 190 comparisons among all individuals. Sub-sampled data points from one speaker were compared to data points from another speaker. The factor *speaker* was considered in all cases as the independent variable, whereas the speech timing parameters deriving from their production were treated as the dependent variable.

Following the comparison of all subjects in the study (i.e., 190 cross-pair comparisons, including the comparison of twin pairs), intra-twin pair differences were identified and systematically reported separately. It is worth mentioning that such a comparison is already expected to yield a great deal of inter-speaker similarity, given that, by taking part in the same dialogue, twin pairs may naturally be under some level of prosodic entrainment, which may, in part, account for their possibly high congruence. It is noteworthy that intra-twin pair comparisons are expected to bear a very low weight on the number of cross-pair differences, given that such a comparison type represents only 5% of all comparisons performed, i.e., 10 out of 190.

The main justification for including the comparisons carried out among all individuals (hereafter, cross-pair comparisons) regards the fact that such data can be regarded as more realistic from a forensic phonetic standpoint, in which individuals may be similar regarding several aspects– such as sex, age, dialect, education degree– but rarely as similar as identical twins. Additionally, in all cases, twins were compared to other twins while interacting with someone they were accustomed to (i.e., their own siblings). As such, the variable inter-speaker “familiarity” may be regarded as equally controlled.

Effect size estimates were computed for all tested parameters based on the comparisons among all individuals (cross-pairs). Such a metric adds to the understanding of how much of the observed variation can be attributed to speaker identity. For the estimation of the Kruskal-Wallis Effect Size, the following formula was applied, where H is the value obtained in the Kruskal-Wallis test; k is the number of groups (i.e., speakers); n is the total number of observations:

$$\begin{array}{c} \eta^{2}=\left(H-k+1\right)/\left(n-k\right) \end{array}$$ (1)

Furthermore, the magnitude of the differences were attributed automatically by the package ‘rstatix’, version 0.6.0, in the R software, in view of the values commonly reported in the literature for the eta-squared (*η*^2^):0.01 ≤ 0.06 (small effect), 0.06 ≤ 0.14 (moderate effect), and ≥ 0.14 (large effect). As such, the effect size index assumes values ranging from 0 to 1, which when multiplied by 100% indicates the percentage of variance in the dependent variable explained by the independent variable, cf. [37, 38].

#### Down-sampling procedure

Given the complex nature of unscripted speech, as expressed by the emergence of different lexical items, phrase length, syllable structures, and different proportions of speech material produced by the subjects, an n-size compensation was performed using a down-sampling procedure.

The referred procedure consisted of randomly sampling a data set so that all classes have the same frequency as the minority class. As a result, all individuals will present the same number of data points, yielding an n-size balanced data set. The primary justification for employing such a procedure lies in the necessity of reducing the discrepancy concerning the number of observations across parameters and speakers, particularly of those that are naturally expected to yield a higher number of observations (e.g., V-V units and vowel segments). Once data sampling is a random process, such a procedure was repeated from 3 to 20 times, allowing the check of consistency concerning possible intra-twin pair differences. It should be noted that tests were run independently, in the sense that significant differences observed during 10 replications, for instance, were not included in the new trial involving 20 replications.

#### The speaker discriminatory performance of speech timing parameters

With regard to the desired properties concerning candidate parameters to speaker comparison ends, two widely acknowledged features– among others– have been suggested, namely, low within-speaker variability and high between-speaker variability, cf. [39].

For such an assessment, three discriminatory performance estimates were examined in the present study to probe the suitability of speech timing parameters for speaker comparison purposes. The first estimate is the *Log-likelihood-ratio-cost function* (Cllr), an empirical estimate of the precision of likelihood ratios proposed by [40], and applied, among others, by [41]. It is given by the Formula 2, in which *Nss* and *Nds* are the number of same-speaker and different-speaker comparisons, and *LRss* and *LRds* are the likelihood ratios derived from same speaker and different speaker comparisons. A same-origin penalty value is *log*_2_(1 + 1/*LRs*), and a different-origin penalty value is *log*_2_(1 + *LRd*):

$$\begin{array}{c} C_{llr}=\frac{1}{2}(\frac{1}{N_{ss}}\sum_{i=1}^{N_{ss}}{\text{log}}_{2}(1+\frac{1}{LR_{ss_{i}}})+\frac{1}{N_{ds}}\sum_{j=1}^{N_{ds}}{\text{log}}_{2}(1+LR_{ds_{j}})) \end{array}$$ (2)

According to [42], such an estimate has the desired properties of being based on likelihood ratios, being continuous, and more heavily penalizing worse results (i.e., providing less support for the consistent-with-fact hypothesis or more support for the contrary-to-fact hypothesis). For computing such an estimate, likelihood ratios were calculated through Multivariate Kernel Density analysis—MVKD [43] (i.e., a non-parametric approach), implemented in the R package “*fvclrr*”, cf. [44]. Multiple pairwise comparisons were performed across individuals in which the background sample consisted of data from all speakers, except those being directly compared (i.e., cross-validation). Likelihood ratios are calculated using the Formula 3, where, as described by [45], LR is the likelihood ratio; E is the evidence, i.e., the measured properties of the voice on the questioned-speaker recording; p(E/H) is *the probability of E given H*; respectively Hs is the same-speaker hypothesis, and Hd is the different-speaker hypothesis (i.e., same-origin and different-origin hypotheses):

$$\begin{array}{c} LR=\frac{p(E|H_{S})}{p(E|H_{d})} \end{array}$$ (3)

Furthermore, a *calibration* procedure was performed, which is a method performed on log-likelihood ratios to reduce the magnitude and incidence of likelihood ratios known to support the incorrect hypothesis, i.e., the contrary-to-fact hypothesis, thereby improving accuracy. Such a procedure is based on a logistic regression model trained with the same set of data (i.e., self-calibration), cf. [42, 45]. For comparability, both calibrated (Cllrcal) and non-calibrated (Cllrraw) metrics are provided. Such a procedure is also implemented in the R package “*fvclrr*”, cf. [44].

The second estimate is the *Equal Error Rate* (EER), which represents the point where the false reject rate (type I error) and false accept rate (type II error) are equal, being used to describe the overall accuracy of a biometric system [46]. Such a metric is also generated along with the Cllr metric using the R package implemented by [44].

Finally, in order to observe the performance of acoustic parameters in terms of their binary classification power, *Receiver Operating Characteristics* (ROC) graphs were plotted. ROC plots are two-dimensional graphs that depict relative tradeoffs between benefits (*true positives*) and costs (*false positives*), providing an estimate that allows the comparison across models/metrics: the “*Area Under the ROC curve*” (AUC) estimate, cf. [47]). Moreover, the multi-class ROC function as defined by [48] was applied to compute the multi-class AUC, which consists of an averaging of multiple AUC estimates. Because of the mathematical solution applied, no ROC curve can be visualized for multi-class AUCs.

An ideal metric for the forensic application should present relatively low Cllr and ERR while displaying relatively high AUC values in relation to the other parameters under comparison. It is noteworthy that some level of correlation between these metrics and the effect size is expected. The higher explanatory potential of the factor speaker on the parameters being assessed is also expected to result in higher inter-speaker separability. Therefore, effect sizes were also taken into consideration in the interpretation of the outcomes.

## Results

Overall, 851 speech chunks were analyzed, an average of 42 chunks and 2.30 min of transcribed material per subject, resulting in an average of 45.5 speech rate and articulation rate data points per subject. Regarding the total length of transcribed material used in the present study, the experiment carried out by [14] with different linguistic units (phone, syllable, V-V units, and word) suggests an average stability time for speaking rate parameters of 12.1 seconds, in which vowel-to-vowel units (V-V units) was the linguistic unit yielding the shortest stabilization time (9.44s). This outcome supports that the average length of the recordings used in the present study is somewhat representative, at least ten times longer than the specialized literature recommends.

For the sake of a general description, median, mean, standard deviations, and range values are presented in Table 1, and the results derived from the statistical analysis summarized in Tables 2–4. Total numbers of observations for each tested parameter are also depicted in Tables 2 and 3.

**Table 1:** Parameters’ categories followed by median, mean, standard deviation and range (of individual means) across subjects.

Parameter	Category	Median	Mean	Standard deviation	Range (means)
SRATE	Macro	4.6 vv/s	4.6 vv/s	1.3 vv/s	3.5—5.7 vv/s
ARTRATE I	Macro	5.5 vv/s	5.4 vv/s	1.1 vv/s	4.7—6.2 vv/s
ARTRATE II	Macro	6.0 vv/s	5.9 vv/s	1.0 vv/s	5.2—6.6 vv/s
SGDUR	Macro	1.0 s	1.2 s	702 ms	853—1.687 ms
VVDUR I	Micro	160 ms	207 ms	199 ms	168—264 ms
VVDUR II	Micro	150 ms	163 ms	98 ms	137—205 ms
VOWEL DUR	Micro	67 ms	84 ms	67 ms	69—104 ms
SILENT PAUSES	Pause-related	480 ms	547 ms	333 ms	398—772 ms
FILLED PAUSES	Pause-related	255 ms	298 ms	146 ms	204—373 ms
ALL PAUSES	Pause-related	365 ms	449 ms	301 ms	345—649 ms
IPI	Pause-related	2.0 s	2.3 s	1.3 s	1.4—3.5 s

**Table 2:** Number of data points, *p*-value and *χ*^2^ for the Kruskal-wallis test (df = 19), number of significant differences among all speakers and intra-twin pairs (Dunn’s Multiple Comparison Test, df = 19, two-tailed test, *p* < 0.025 with Bonferroni adjustment), followed by effect sizes (*η*^2^).

Parameter	N	*p*-value/*χ*^2^ (cross-pairs)	Cross-pair differences	Intra-twin differences	Effect size (cross-pairs)	Magnitude (cross-pairs)
SRATE	851	< 0.001/148.7	40 (21.0%)	–	15.6%	Large
ARTRATE I	851	< 0.001/147.8	47 (27.7%)	–	15.5%	Large
ARTRATE II	851	< 0.001/121.3	26 (13.6%)	–	12.3%	Moderate
SGDUR	2.107	< 0.001/156.5	42 (22.1%)	–	6.5%	Mod
VVDUR I	12.609	< 0.001/305.0	75 (39.4%)	G1-G2	2.2%	Small
VVDUR II	10.495	< 0.001/268.3	62 (32.6%)	G1-G2	2.3%	Small
VOWEL DUR	9.447	< 0.001/183.5	54 (28.4%)	J1-J2	1.7%	Small
SIL PAUSES	864	< 0.001/58.2	7 (3.6%)	C1-C2	4.6%	Small
FIL PAUSES	560	< 0.001/64.8	10 (5.2%)	–	8.3%	Moderate
ALL PAUSES	1.424	< 0.001/66.9	7 (3.6%)	–	3.3%	Small
IPI	675	< 0.001/92.8	21 (11.5%)	F1-F2	11.3%	Moderate

**Table 3:** Number of significant differences (Dunn’s Multiple Comparison Test, df = 19, two-tailed test, *p* < 0.025 with Bonferroni adjustment) in intra-twin pair comparisons for VVDUR I, VVDUR II, VOWEL DUR, and SIL PAUSES for downsized samples.

Parameter	N	Random sampling Intra-twins 3 replications	Random sampling Intra-twins 10 replications	Random sampling Intra-twins 20 replications
VVDUR I	8.580	G1-G2 (1x)	G1-G2 (1x)	G1-G2 (4x)/J1-J2 (1x)/E1-E2 (1x)
VVDUR II	7.020	–	G1-G2 (1x)	G1-G2 (2x)
VOWEL DUR	8.040	–	J1-J2 (7x)	J1-J2 (8x)
SIL PAUSES	440	–	C1-C2 (1x)/ A1-A2 (1x)	C1-C2 (3x)

**Table 4:** Raw and calibrated likelihood-cost ratios (Cllr), equal error rates (EER), multi-class AUC values for cross-pair comparisons, and effect sizes (*η*^2^).

Parameter	Cllr_raw_	Cllr_cal_	EER	AUC	Effect size
SRATE	0.78	0.78	0.28	0.64	Large
ARTRATE I	0.76	0.75	0.27	0.64	Large
ARTRATE II	0.78	0.75	0.31	0.62	Moderate
SGDUR	0.96	0.89	0.35	0.59	Moderate
VVDUR I	0.82	0.81	0.33	0.55	Small
VVDUR II	0.92	0.84	0.30	0.55	Small
VOWEL DUR	0.95	0.90	0.40	0.54	Small
SIL PAUSES	6.06	1.00	0.55	0.58	Small
FIL PAUSES	2.81	1.00	0.50	0.61	Moderate
ALL PAUSES	9.97	1.00	0.50	0.56	Small
IPI	0.88	0.88	0.43	0.63	Moderate

### Intra-twin pair comparisons

As can be seen in Table 2, the analysis of speech timing patterns in comparisons involving identical twins revealed a remarkable level of intra-pair similarities. This observation applies particularly to the class of macro speech timing parameters, for which the largest effect sizes were observed for the general population, except for one estimate: SGDUR. As for micro temporal parameters, two pairs out of ten (20%) were found statistically distinct, namely G1-G2 and J1-J2. The same proportion of intra-twin pair differences can also be observed for the class of pause-related estimates, with two pairs diverging significantly: C1-C2 and F1-F2. As may be seen in Table 2, apart from IPI, all temporal parameters pointing to intra-twin pair divergences were found to display “small” effect sizes as a function of the speaker identity. Based on such an observation, a re-test was conducted with down-sampled data points of the respective parameters.

Intra-twin pairs and cross-pair statistically significant differences after the down-sampling procedure are presented in Table 3, based on three random samplings. As can be observed, when reducing the number of observations to about 32%, 33%, 15%, 49%, for VVDUR I, VVDUR II, VOWELDUR, and SILPAUSES, respectively, and repeting the procedure, no consistent differences could be verified for the respective speech timing estimates. In contrast, when several independent replications are performed, twin pairs that could not be contrasted earlier stood out as significantly different, as verified for VVDUR I and SILPAUSES in Table 3. Such a lack of consistency may serve as a clue to a probable n-size or sampling influence on the outcomes.

Finally, when disregarding all reported inconsistent differences, only F1-F2 turns out as statistically different for IPI with an AUC of 75%. However, the level of robustness of this parameter for the forensic phonetic application, as far as its discriminatory performance is concerned, is arguable, as will be described further.

**Fig 2:**
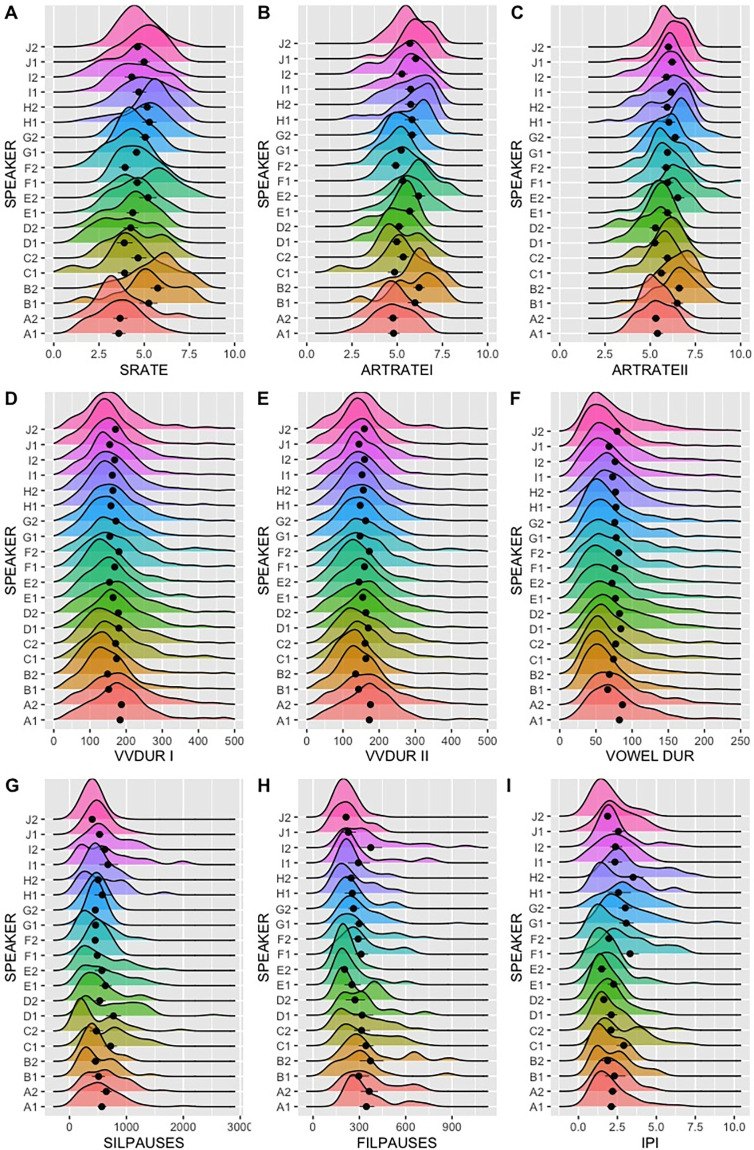
Diagram of density for speech rate (A), articulation rate I (B), articulation rate II (C), V-V unit duration I (D), V-V unit duration II (E), Vowel duration (F), silent pauses (G), filled pauses (H), IPI (I).

In Fig 2 density curves are presented for all temporal parameters and all speakers according to the Kernel density estimate, which may be regarded as a smoothed version of the histogram. Through a close inspection of this figure, it is possible to see how similar identical twins were regarding their speech timing patterns, as expressed by the relative overlap of their density curves. It can be seen that they are almost perfectly aligned in terms of their mean values, to a greater extent for micro speech timing estimates and to a lesser extent for pause-related estimates. Conversely, when comparing the density curves across all speakers, some differences can be observed, mainly at a macro speech timing level.

### Cross-pair comparisons

All results deriving from the cross-pair comparisons are also summarized in Table 2. In the table, cross-pair significant differences are expressed as total values and percentage values, considering the proportion of differences observed as a function of the number of cross-pair comparisons performed (i.e., 190 cross-pair comparisons).

As can be seen, there was a tendency for those units with a higher frequency of occurrence to display a higher number of inter-speaker differences than in micro speech timing parameters. The second parameter category displaying the highest proportions of inter-speaker differences was the class of macro speech timing parameters. Finally, parameters pertaining to the class of pause-related estimates was found to display the lowest proportions of inter-speaker differences, suggesting a higher convergence for such estimates across speakers, especially for silent pauses (SILPAUSES).

When comparing the effect size values presented in Table 2, which is an estimate that provides a common metric to compare the direction and strength of the relationship between variables [9], it is possible to observe how much of the variation for each tested parameter can be explained on the basis of the “speaker” variable, as expressed by the effect sizes comparisons across different parameters. Hence, effect sizes can be regarded as an important indication of whether the differences observed are likely to be explained on account of individual differences or better explained by other factors.

Considering the magnitude of the effect sizes presented in Table 2, two speech timing parameters were found the most explanatory of individual patterns, namely speech rate, and articulation rate I (i.e., excluding silent pauses). As far as the explanatory potential of the speaker identity is concerned, these two parameters were virtually identical in terms of explanatory power. However, when considering the proportion of inter-speaker differences, a slightly higher proportion of differences was noted for ARTRATEI in relation to SRATE. Moreover, slightly smaller effect size and proportion of inter-speaker differences were observed for ARTRATE II (i.e., excluding both silent and filled pauses) in relation to SRATE and ARTRATEI. Note that these are the only parameters based on the same number of observations, making their comparison less biased.

Ordering the explanatory potential of the speaker identity from the largest effect to the smallest effect of the variable on the different speech timing parameters we arrive at the following order:

$$\begin{array}{c} \text{SRATE}=\text{ARTRATE}\;\text{I}\\ >\;\text{ARTRATE}\;\text{II}\\ >\;\text{IPI}\\ >\;\text{FILPAUSES}\\ >\;\text{SGDUR}\\ >\;\text{all}\;\text{the}\;\text{other}\;\text{parameters} \end{array}$$

By comparing overall patterns of the density curves in Fig 2 and individual mean values as a function of all parameters, it can be observed how variable speech timing estimates are across speakers, also helping to understand their effect size differences.

### The speaker-discriminatory performance of speech timing parameters

One of the goals of the present study was to identify the most suitable speech timing parameters for speaker comparison applications from a forensic perspective, which regards not only how variable estimates are across individuals but also how accurate and consistent they might be. Three different estimates were used to test such a consistency: the Log-likelihood-ratio-cost function (Cllr), Equal Error Rate (EER), and AUC values deriving from multiple ROC analyses. In Table 4 performance estimates are presented for each analyzed parameter. For comparability, effect size magnitudes are re-evoked. Observed Cllr and EER values are reported after performing several tests and averaging the results.

As can be seen in Table 4, among all tested parameters, SRATE and ARTRATE I, both from the class of macro temporal parameters, have shown to display the largest AUC values and the lowest EER. These were also the parameters displaying the lowest Cllr values, along with ARTRATE II. From this parameter category, SGDUR presented the worst overall performance with EER around 35%.

Concerning the category of micro temporal parameters, these were found to exhibit the lowest AUC values of all tested estimates, with an overall classification performance just above the chance level (54%-55%), being outperformed by all macro temporal estimates. VOWEL DUR showed the worst speaker-discriminatory performance from this parameter group, expressed by the highest Cllr/EER and the lowest AUC values.

Finally, by inspecting Table 4, it can be seen that category of pause-related parameters exhibited the highest Cllr and EER values, with SILPAUSES and ALL PAUSES (i.e., the combination of silent and filled pauses) displaying the worst discriminatory performances, as evidenced by the highest EER and Cllr values among all tested parameters, even when considering calibrated Cllr values. As can be noted, from this parameter class, IPI was found the best performing parameter; however, with equally high EER values: 40%-45%.

Note that, in terms of Cllr, the pause-related parameters category was the one that benefited most from a calibration procedure, as expressed by a considerable reduction between raw and calibrated Cllr values. However, their Cllr values were still very high (equal or close to 1), suggesting a poor performance. The other parameters did not benefit as much from such a procedure using the present data configuration.

**Fig 3:**
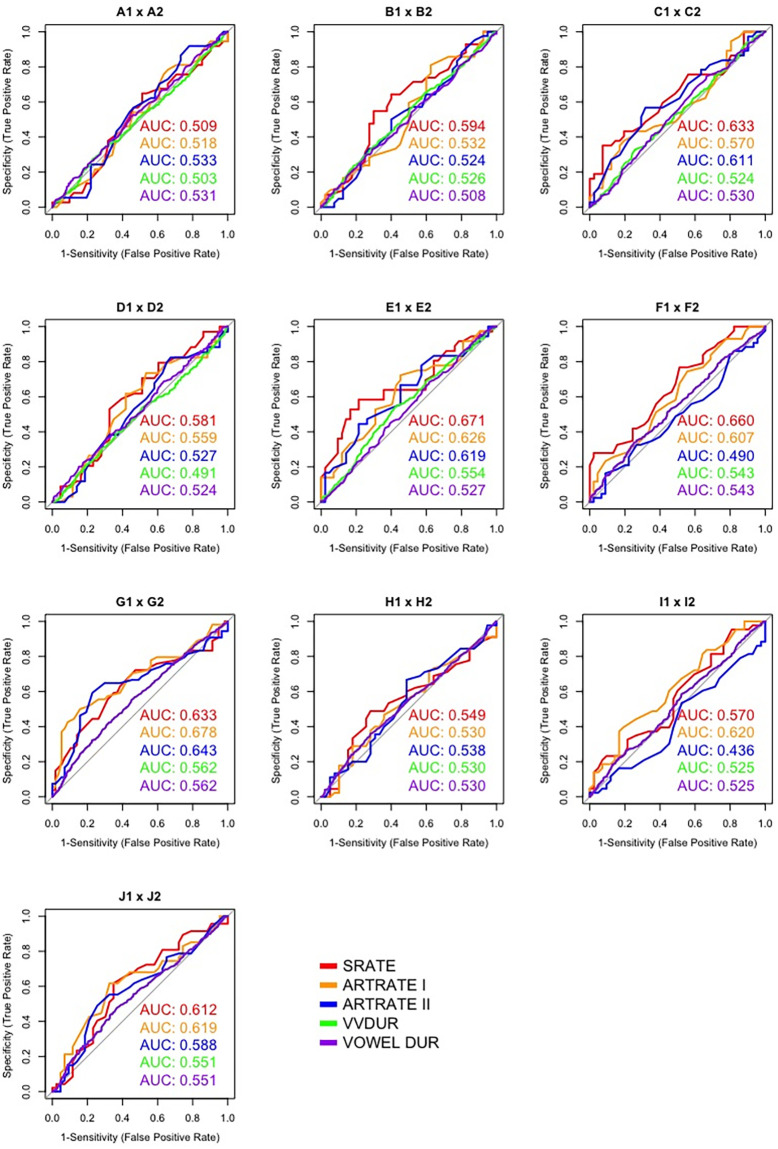
ROC curves and AUC values for intra-twin pair comparisons.

**Fig 4:**
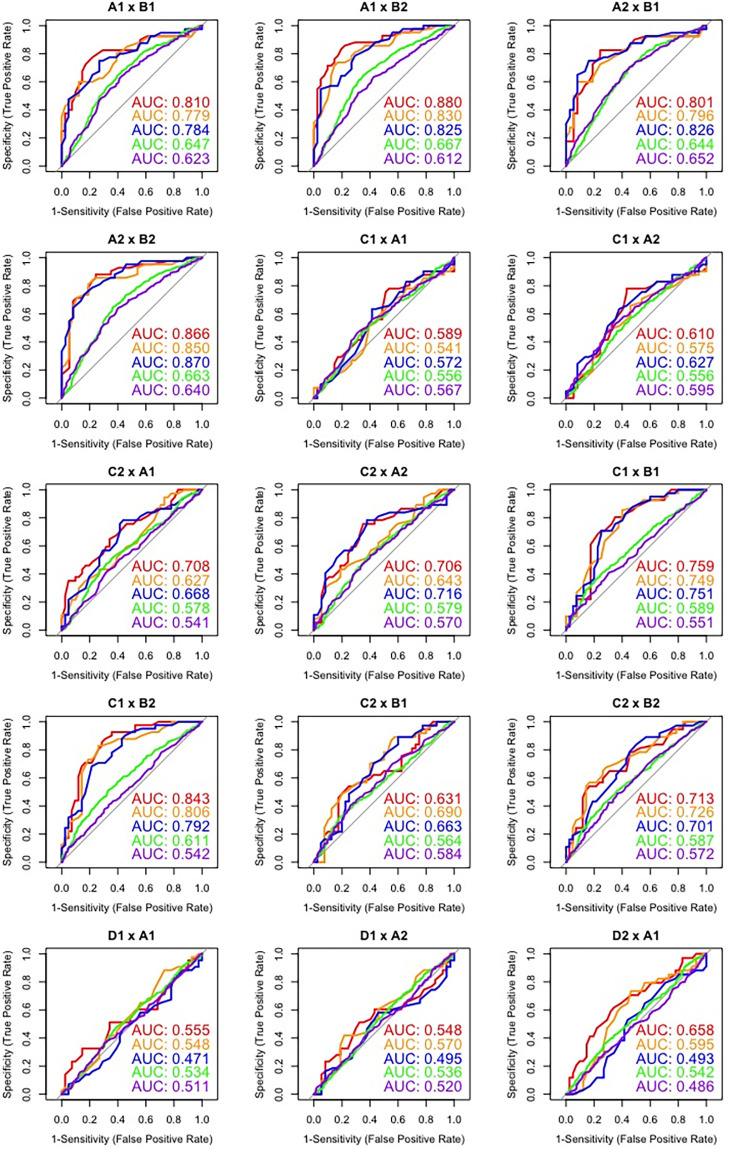
ROC curves and AUC values for cross-pair comparisons (I).

**Fig 5:**
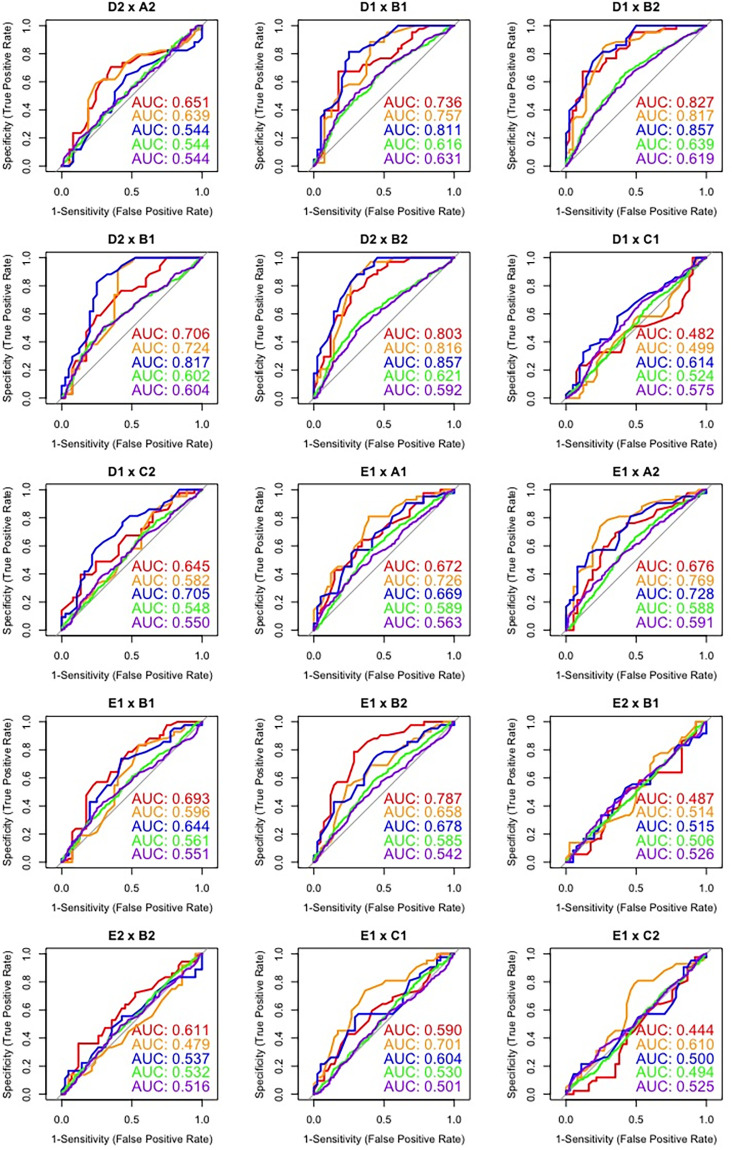
ROC curves and AUC values for cross-pair comparisons (II).

ROC graphs corresponding to intra-twin pair comparisons are displayed in Fig 3, while cross-pair comparisons regarding the first ten speakers are presented in Figs 4 and 5. Such plots depict the overall classification performance regarding the most discriminatory macro speech timing parameters (i.e., SRATE, ARTRATEI, ARTRATEII) in relation to VVDUR I and VOWEL DUR. For the sake of simplicity, the other parameters were not included; however, their overall performance can be assessed in Table 2. Such figures show an important feature that is not represented in Table 2.

By inspecting Fig 3, it is possible to verify that, as anticipated, intra-twin pair comparisons by means of ROC analysis yielded, overall, a very poor classification performance, expressed by AUC values just above the chance level (50%) for some estimates, and even below the chance level for others. Notwithstanding, regarding cross-pair comparisons, Figs 4 and 5 show that there was no homogeneous discriminatory performance for any of the parameters assessed across different pairs. On the contrary, substantial performance differences were observed on account of the pairs being compared.

Furthermore, when comparing the performance of macro and micro speech timing parameters in Figs 4 and 5, it is possible to note that, in the large majority of cases, macro speech timing estimates presented overall better performances than micro speech timing estimates. It can also be observed that even across individuals that are not taking part in the same dialogue and who are not genetically related, very poor classification performances can be observed on account of their speech timing patterns (e.g., D1 x A1; D1 x A2; E2 x B1; E2 x B2). This relatively high variation in classification performance across different pairs of speakers may be regarded as the main reason for such low global AUC values in Table 4, since the reported multi-class AUC values consist of the averaging of multiple AUC values, including average, above-average and below-average discrimination performances.

Finally, it is worth mentioning that the poor performances observed for intra-twin pair comparisons are expected to bear a very low weight on the global AUC values reported in Table 4, since this kind of comparison represents 5.2% of all comparisons performed, namely 10 out of 190 inter-speaker comparisons.

## Discussion

The present study set out to assess the speaker-discriminatory potential of a set of 11 speech timing parameters in comparisons performed with identical twin pairs and cross-pair comparisons. Speech timing estimates pertaining to different dimensions were assessed; namely, macro, micro, and pause-related, having the phonetic syllable duration as the main criterion for contrasting the first two categories. The outcomes are discussed in the following.

### The discriminatory performance of macro, micro, and pause-related speech timing parameters

From a general perspective, the present research findings suggest the category of macro speech timing parameters as the most reliable estimates when assessed under unscripted speech conditions, mainly SRATE and ARTRATE II. Furthermore, a relatively similar speaker-discriminatory performance has been observed regarding the comparison made between the two estimates, with ARTRATE I displaying comparable Cllr/EER values and a relatively higher number of inter-speaker differences. As for ARTRATE II, which is characterized by the suppression of both silent and filled pauses during its calculation, a slightly lower discriminatory performance was suggested. In addition, SRATE and ARTRATE I were also the estimates presenting the largest effect sizes, which is compatible with a higher explanatory power of the speaker identity in their variation patterns.

However, it is worth noting that, despite yielding the best performing parameters, the overall performance of SRATE and ARTRATEI was found relatively poor when assessed in isolation, adding some uncertainty whether those parameters would provide enough support for the application in actual forensic conditions, as will be commented on in the upcoming sections.

As for micro speech timing parameters, despite their considerably higher number of cross-pair statistical differences, this was the category displaying the smallest effect sizes concerning all tested estimates as a function of the “speaker” factor. Moreover, contrary to the present authors’ expectations, the reported effect size did not appear to be largely dependent on the V-V units’ salience, as defined by means of a duration criterion. Such an outcome invites other explanatory factors regarding micro speech timing parameters’ variability, such as factors of a linguistic order.

In this regard, some variables have been systematically reported to significantly affect syllable duration across languages, such as stress and syllables’ position in the phrase. In this context, duration has been acknowledged as the most reliable explanatory factor of stress across different languages [49], with stressed vowels (i.e., the nucleus of the syllable) displaying longer duration in comparison to unstressed vowels, as in BP [50]. As for the longer duration of syllables in a phrase final position, the so-called “phrase-final lengthening effect”, is a widely reported phenomenon occurring to the final syllable rime [51]. Moreover, studies have shown that, although most of the duration increase seems to occur in the phrase-final syllable rime, significant lengthening has also been found in the main-stress syllable rime, when this is not the final syllable, as observed by [52] for American English. Another potentially relevant explanatory factor regards the presence of pauses within the bounds of V-V units, which is assumed to result in a duration increase; such duration increase is associated with phrasal prominence throughout utterances [11]. In this regard, the duration of pauses, either their presence or absence within the bounds of V-V units, may be a better explanatory component regarding the variability of these units. However, adding or subtracting such phenomena from V-V units did not seem to substantially increase the explanatory potential of the factor “speaker” on their variation.

As for vowel duration (VOWEL DUR), a low explanatory potential of the speaker identity has also been observed, which, similarly to the duration of V-V units, may likely be better accounted for by linguistic constraints. As remarked by [53], studies of vowel duration have resulted in two well-known general formulations, that is, the duration of a vocalic segment is largely related to the degree of opening of the vowel, resulting in a higher duration for low vowels in comparison to high vowels, and that its duration also depends on the nature of the following consonant, with vowel segments being longer before voiced and shorter before voiceless consonants. Together, the before-mentioned factors may explain, in part, why individual variability seems to display a low explanatory potential concerning the duration of micro speech units.

The fact that a higher number of inter-speaker differences have been observed for the category of speech timing estimates does not imply that such differences can be attributed to idiosyncratic patterns. Such an outcome may be related to the fact that vowels and syllables are relatively more frequent than larger speech units (e.g., words, phrases), yielding a greater number of data points reflecting on the statistical power of the analysis, allowing the detection of very small differences. Whether such differences are linguistically meaningful must, however, be determined independently. In this regard, as pointed out by [9], while p-values are highly influenced by sample size and more likely to be significant when the sample size is large and less likely if the sample is small, effect size estimates, in contrast, are not sensitive to it. The outcomes of the present study seem to present clear support for this statistical fact.

### Some remarks on speaking rate

The observation that ARTRATE I was the parameter displaying the largest proportion of inter-speaker differences within the category of macro speech timing parameters is in broad agreement with the widespread assumption of articulation rate as considerably speaker-specific [8, 17]. Notwithstanding, it must be noted that different outcomes may be observed depending on the nature of the articulation rate under assessment, as to whether only silent pauses or both silent and filled pauses are excluded. When estimating the articulation rate by considering both silent and filled pauses (i.e., ARTRATE II), a reduction in the proportion of significant differences across speakers was observed. A basis for such a reduction may be suggested, namely the exclusion of idiosyncratic information conveyed by voiced segments, particularly of filled pauses. As pointed out by [17], the speaker-specific potential of filled pauses is well known, following the observation that individuals tend to be quite consistent in using ‘their’ respective personal variant of the hesitation sound. In the present study, filled pauses displayed a moderate effect size, being suggested as more explanatory of individual patterns than silent pauses. It must be recognized that, as pointed out by [13], the identification and transcription of filled pauses is a somewhat laborious task often requiring several revisions. In some cases, it is uncertain whether one is dealing with a filled pause, an emphasis, or perhaps both, expressed in the form of a lengthened vowel.

In our study we observed that the difference in the speaker-discriminatory potential between articulation rate and speech rate seemed dependent on the treatment given to silent and filled pauses. Similar observations of the discriminatory potential regarding the two parameters have been suggested in the available literature. Despite observing a higher variance for speech rate in relation to articulation rate, [13] noted a compatible discriminatory power between speech rate and articulation rate in an experiment with BP speakers. However, the before-mentioned study differs from the present regarding some essential aspects. Firstly, the number of speakers analyzed, 20 in the present in relation to 7 speakers in the other. Secondly, for the articulation rate estimation, not solely silent pauses were excluded from the intervals’ total duration, but also filled pauses. As such, the articulation rate reported by that author is somewhat equivalent to ARTRATE II in the present experiment, which appears to be the case when comparing the global (6,19 vv/s) and local (6.20 vv/s) median values reported by [13] with the median value observed here (6 vv/s). Thirdly, the minimum silent pause duration threshold in the present study was set at 100 ms in comparison to 130 ms on the other, which may result in different estimates. These factors combined, adding that different assessment criteria were adopted, may account for possible cross-study differences.

As previously mentioned, the category of macro speech timing parameters was the best explained on account of the speaker identity. This is particularly true for SRATE, ARTRATE I, and ARTRATE II, which are measures extracted from larger temporal windows (see Table 2). Such an outcome suggests a rather interesting tendency: the effects of individual variation in speech timing parameters seem more expressive in larger temporal intervals than in small temporal windows. This trend may find support in the observation that speakers can vary significantly in the proportion of silent or filled pauses they produce, or even in the proportion of lengthening in word-final segments, as mentioned by [17], depending on different factors, such as speaking style and emotional state. However, the same “*freedom*” does not seem to apply to smaller units, such as syllable duration or vowel duration, where a great deal of individual variability could have consequences on communication or on the intrinsic rhythm structure. Additional evidence of a higher individual (idiosyncratic) articulatory control on macro over micro temporal units may be provided by the observation of a higher agreement across individuals for macro speech units than for micro in the production of synchronous speech [54]. It is worth noting that such a speaking condition is regarded as having direct consequences on the prosodic variability, reducing idiosyncratic and expressive variation across individuals [55].

With regard to average values concerning some of the most commonly studied speech timing estimates, such as speech rate and articulation rate, the average values observed in the present study are in some agreement with values reported in the literature for spontaneous speech. In this regard, average values ranging approximately from 4 to 5 syl/s for speech rate and from 5 to 6 syl/s for articulation rate have been reported across different studies, cf. [8, 13, 17, 56, 57]. Such a convergence should not be regarded as arbitrary, as it suggests a regular pattern across different languages [58]. Moreover, the results recently obtained by [59] provide empirical evidence based on studies at the cortical level that there seems to be a preferred speaking rate based on neuronal processing. By measuring the synchronization between auditory and speech-motor regions in the brain while participants listened to synthesized syllables at different rates, the authors of [59] found that the auditory-motor synchrony was significant only over a restricted range, being enhanced at 4.5 Hz. According to the researchers, this is a value compatible with the mean syllable rate across different languages (for a deeper understanding, see also [60]). According to [59], these findings suggest that the temporal patterns of speech emerge as a consequence of the intrinsic rhythms of cortical areas, yielding a reliable coupling between acoustic stimuli and auditory cortical activity. In the present study, the observed mean speech rate across speakers lied within this referred interval, where a mean/median speech rate of 4.6 vv’s/s and a standard deviation of 1.3 vv’s/s were observed.

Several other studies also support the observation that neural activity phase-locks to rhythm, not only in speech but also in music [61–64]. In a literature review by [58], the researchers explored studies with what they call the ‘temporal mesoscale’ of speech, with special attention to regularities in the envelope of the acoustic signal that correlate with syllabic information. It has been observed that the temporal structure of speech at this scale is remarkably stable across languages, with a preferred range of rhythmicity of 2– 8 Hz. As argued by the authors of [58], this rhythmicity is required by the processes underlying the construction of intelligible speech. The relevance of the outcomes in the referred studies when interpreting the findings in our study is that they seem to concomitantly signal the limits of variability expected for the *rate of speech*, based on an intertwined relation between production and perception. In that sense, although speakers do tend to vary in their speech temporal patterns, the magnitude of this variation may be seen as dependent on production-oriented and output-oriented constraints, driven by demands of production efficiency on the one hand, and comprehensibility on the other, as thoroughly exploited in [65].

### Limitations of measuring speech timing for speaker comparison ends

While inspecting estimates in Table 2, one has to admit that, overall, the discriminatory performance of speech timing estimates may be considered relatively poor. This is particularly the case when comparing to other acoustic parameters, such as melodic metrics, cf. [66]. In this instance, previous research has acknowledged the relatively poorer discriminatory potential of speech timing estimates. While assessing the discriminatory power of speech timing parameters, including speech and articulation rates, [17] remarked that, through the verification of their equal error distributions, it is necessary to acknowledge that the discriminating capacity of speech timing measures seemed rather poor in comparison with other acoustic estimates, such as linear predictive coding (LPC) or cepstral coefficients. Despite such an observation, the author of [17] also emphasizes that an estimate such as articulation rate is much more appropriate for use under real-world forensic conditions, often involving telephone transmitted speech.

Moreover, in the experiment conducted by [67] with 30 English speakers, aiming to compare common speaking rate measures (e.g., rates based on the counting of canonical and surface syllable, phones, and CV segments), it was verified that these rates were closely inter-correlated yielding similar discriminating powers. Notwithstanding, as remarked by the researchers, the results suggested that tempo is a relatively poor speaker discriminant regardless of methodology, as characterized by rather high EERs and Cllrs close to 1.

The analyses carried out by [57] on the implication of reference sample size, and the calculation of numerical LR based on articulation rate revealed the same tendency. In the referred study, both EER and Cllr average values were found relatively high—35% and 0.97, respectively– suggesting an overall poor performance of articulation rate for forensic speaker comparison. Furthermore, it was verified that the EER estimate tended to remain stable/consistent with the increase in the number of tokens, not presenting important repercussions in terms of categorical system validity. As for Cllr, calibrated LRs were found to be robust to sample size effects, whilst non-calibrated scores displayed much more sensitivity to the amount of reference data used.

Notably, the effects of speaker/data sampling on EER and mostly on Cllr values must be considered in future developments of the present study as to obtain relevant information on system stability regarding the parameters assessed and the level of uncertainty concerning the LR computation, cf. [68, 69].

### Differences in pause-related parameters

Notably, within the category of pause-related parameters, the inter-pausal intervals (IPI) have shown to display the highest number of significant differences across individuals, as well as the largest effect sizes (see Table 2). These finding are in good agreement with [20], mentioning that there is considerable variation in the manner that individuals convey a message, including frequency of pauses which determine the length of inter-pausal chunks of fluent speech. In that study, the author observed a considerable inter- and intra-speaker variation in producing interpausal phrases.

The observation that silent pause duration yielded the fewest number of differences in relation to all the remaining parameters in the present study seems to suggest a relative regularity of pause-related measures across individuals. This outcome is no surprise when considering the key role played by silent pauses in revealing the prosodic structure of utterances, with its emergence being commonly associated with intonational phrase boundaries [70]. In that regard, in the study conducted by [71], aiming to assess the effect of prosodic structure and phrase length on pause duration in read sentences, the researcher was able to observe a pre- and post- boundary prosodic effect on silent pause duration, in which longer phrases, both before and after the phrasal boundary, yielded longer pauses. Moreover, the researcher was able to identify a prosodic complexity effect on pause duration, in which medial prosodic boundaries induced shorter pauses in comparison to final boundaries. In the same direction, [72] was able to observe pause-duration related differences as a function of structural factors concerning the discourse organization. It was also noted that speakers tended to display longer pauses at topic shift than at other discourse boundaries, which also seemed to influence the amount of sentence-final lengthening.

Based on the findings in the literature, a point of convergence can be identified across studies. Silent pause duration, as opposed to its frequency, seems to be largely dependent on intrinsic factors, which may in part suggest a substantially high linguistic control on its variability in communicative contexts. The present study appears to provide evidence for a low inter-speaker variability regarding global measures of pause duration. Additional support for this relative pause duration stability across individuals, may find ground in the observation that the frequency of pauses has been reported to be more variable than its duration, as suggested by [73]. The researchers noted that when speakers vary their rate of reading to produce a desired apparent rate, they primarily tend to add or subtract pauses of largely the same duration at strategic syntactic locations, whereas articulation rate and pause duration are much less affected.

Note, however, that different levels of variability in pausing behaviour can be identified across individuals when a distinction between medial sentence and final sentence pauses is made. In that regard, [74] found evidence in Swedish suggesting uniform patterns of pause duration between complete sentences across subjects, whilst pauses within sentences showed large individual variations in reading. Further developments of the present study must also probe this possible pause-type dependent difference in spontaneous speech.

Finally, it should be acknowledged that “silent pause duration” may be efficient when used for differentiating different speaking styles. In the study conducted by [75] with Polish, on the application of pauses as a potential source of biometry for automatic speaker recognition, three types of acoustic pauses (silent, filled and breath pauses) and syntactic pauses were analyzed in both spontaneous and read speech. The researchers found that quantity and duration of filled pauses, audible breaths, and correlation between the temporal structure of speech and the syntactic structure were the best performing features for speaker characterization. Silent-pause related features, on the other hand, considerably improved the distinction between read and spontaneous speech with 75% accuracy.

### A note on synchronicity in speech production

The findings in the present study provide evidence of speech timing patterns as being remarkably similar within twin pairs. This convergence was considerably stronger than that observed in a previous study on vowel formant frequencies involving the same identical twin pairs and using precisely the same excerpts of speech material, cf. [29]. Two complementary hypothesis are invited to account for such a striking intra-twin pair similarity regarding the temporal dimension: the sharing of similar mental representations of speech timing features acquired throughout their language acquisition and the possible (overlapping) effects of prosodic entrainment.

In this regard, evidence from experimental studies on motor control of articulatory timing at the phoneme [76] and word level [77] provide an indication of motor control of timing at those levels as relatively more “hard coded” than the motor patterns involved in the production of spectral components of speech. The experiment conducted in [76] with the impersonation of phonetic patterns, including the temporal dimension of speech production, revealed that timing patterns in the imitations were in all cases more similar to those of the impersonator’s natural production than to the target patterns (i.e. the speech model to be reproduced), which may suggest that the speech timing features are more challenging to be deliberately manipulated, and perhaps more stable intra-individually. Such observations are of considerable relevance from a forensic-phonetic perspective, as they suggest a relative intra-speaker stability of speech temporal patterns.

Support for an environmental effect on the establishment of speech temporal patterns has been found under a more controlled situation, i.e., read speech. In the study conducted by [4], a pair of male identical twins (T1 and T2) aged 21-years-old and an age- and sex-matched sibling (S), recorded 2 years after, were assessed regarding their speech tempo and fundamental frequency patterns. The authors of the referred study found evidence for greater intra-twin similarities in mean *f*0 compared to the control. Conversely, although intra-twin similarities were greater than the similarities to the siblings, these differences diminished for both speech tempo and dynamic *f*0 parameters at the sentence, word, and syllable level. According to the researchers, such an outcome supports the view that some speech and voice parameters might be under greater genetic influence, whereas other parameters, like accent, dialect, reading style, and speaking style are mostly shaped by environmental factors.

The methodological design adopted in the present study does not allow answering to what extent or to what level the high intra-twin pair temporal congruence may solely be accounted for by environmental influences, since another equally plausible and perhaps overlapping factor is suggested; prosodic entrainment. In addition, the fact that more differences have been found regarding spectral and melodic parameters for the same twins in [29, 66] may indicate a possible higher level of prosodic entrainment in speech timing than in formant frequency and melodic patterns. In the experiment on perceptual entrainment conducted by [78] with 42 BP native speakers encouraging evidence was found supporting the assumption of rhythm perception as a listener-speaker entrainment process, in which duration may be considered the main acoustical feature driving the behavior of listeners. Furthermore, prosodic boundaries (i.e., stress group boundaries) were found to play an important role in such a mechanism, organizing the listener’s experience of rhythm.

The concept of abstract clocks responsible for regulating the temporal organization of motor gestures and consequently allowing individuals to enter into a “*synchronization state*” is not exclusive to the realm of speech production. In fact, such mechanisms have been observed for other complex forms of timing and motor control, e.g., music playing, typing, cf. [79, 80]. As for speech production, several experimental studies have been carried out in the domain of synchronous speech, with particular attention to the research performed by [54, 55, 81–83].

In [54], while assessing two subjects reading a text in synchrony, it was observed that prosodic variability was significantly reduced when reading synchronously. In particular it was demonstrated that synchronous speech exhibited markedly less inessential variability. Moreover, variables associated with global timing, namely major syntactic juncture and phrase length, were found more consistent in the synchronous condition, displaying less variability, while smaller units were not noticeably affected by the synchronous speaking condition, as in the case of stressed and unstressed syllables, as well as the closure to voicing onset (C-V transition). In sum, those variables which were most directly related to macroscopic temporal structure (i.e., phrasing) displayed less variability in synchronous speech. In contrast, the microscopic temporal structure remained stable, suggesting, according to the researcher, that at a finer timescale, there is little if any change to speakers’ timing when speaking in synchrony.

Regarding the effects of interpersonal synchrony when two or more individuals take part in the same conversation, [84] points out that conversational speech by one individual must be patterned to somehow “fit” the patterns of the other individuals engaged in the conversation, referring to effective turn-taking as one example of such fit. Moreover, as mentioned by the researchers, there are other ways in which speech can be temporally structured during conversational interaction. By analyzing the prosodic cycles of mean speech fundamental frequency and mean voice intensity between subjects while engaging in a conversation, [84] observed a common occurrence both in Swedish and American English, namely the relative continuance of speakers’ “rhythms” across turn-exchanges, in which the period and phase of prosodic cycles initiated by one partner was maintained by the other, across speaker transitions. Furthermore, the analyses suggested that in conversations between same-sex speakers, the patterns of rhythmic integration, or “synchrony”, were substantially similar in American English and Swedish.

Such a synchronicity or “*convergence*” between interlocutors has been observed and corroborated by [85] at the speech timing level while keeping under control two possible driving forces related to this convergence, namely, conversational factors and the interlocutor baseline speech rate. According to the authors of [85], such a convergence effect may be either a direct or indirect process, in that the interlocutors’ different speech rates may affect some intermediate factor (e.g., conversation flow) and thereby affect the speakers’ speech rate.

As mentioned by [54], the answer to synchronicity must lie in the shared knowledge speakers have of what is essential and what is redundant, or optional, in the modulation of the speech organs. This observation also concerns speakers of the same dialect, who very likely share similar temporal relations among the discrete speech units and the mechanisms for producing them.

As for genetically related speakers, a great deal has yet to be explored, mainly concerning the effects of acquiring a language “together”, being exposed to very similar models (e.g., same mother, father, and relatives) on the establishment of their “linguistically shared knowledge”, and, consequently, on their speech timing patterns. To that end, data from non-identical twins (dizygotic twins) should be included in future studies, including a different experimental design and the assessment of distinct speaking styles.

## Conclusion

The results in the present study support the observation that the macro speech timing parameters, mainly speech rate and articulation rate, are the most discriminatory and consistent parameters for forensic speaker comparison application under unscripted speech conditions. Although very similar outcomes have been observed regarding the comparison of speech rate and articulation rate, different performance outcomes were observed depending on whether only silent pauses or both silent and filled pauses were suppressed during the calculation of the parameter. In summary, when only silent pauses were suppressed for the articulation rate estimation, a slightly better performance was suggested, as expressed by lower EER and higher AUC values along with a higher effect size. The analysis of speech timing parameters in identical twin pairs, taking part in the same dialogue, revealed a remarkable level of intra-pair similarities, substantially higher than the similarities observed for the same speakers’ formant frequency patterns analyzed in the same speaking condition, as explored in a previous study, cf. [29]. Some explanatory factors, such as “prosodic entrainment”, were suggested and discussed. Moreover, the present study’s findings suggest that the speaker-discriminatory potential of speech timing parameters is far from uniform across speakers; hence their forensic suitability should be assessed on a case-by-case basis and considered with caution. Furthermore, given the homogeneous characteristics of the data assessed and its size, i.e., relatively long speech stretches produced by young male individuals in the same dialect and speaking condition, it is expected that different levels of discriminatory power may be observed in real-life speaker comparison circumstances. Finally, the effects of speaker/data sampling on EER and mostly on Cllr values must be considered in future developments of the present study, as to obtain relevant information on system stability regarding the parameters assessed.
